# Supplementary material for: Explainable artificial intelligence for personalized prognosis in pancreatic cancer: A nationwide study from Taiwan
Source: PLOS Digit Health. 2026 Mar 19;5(3):e0001296. doi: 10.1371/journal.pdig.0001296 (PMC13001956; doi:10.1371/journal.pdig.0001296)
Supplement: S1 Table — (PDF) [file pdig.0001296.s001.pdf]

S1 Table. ICD-O-3 morphology codes for pancreatic cancer histological types.

Histological types	ICD-O-3 morphology codes
Adenocarcinoma	8020, 8035, 8140-8141, 8143-8144, 8148, 8160, 8163, 8210-8211, 8230, 8245, 8255, 8260-8261, 8310, 8323, 8430, 8440-8441, 8450, 8453, 8470-8472, 8480-8481, 8490, 8500, 8503, 8523, 8552, 8560
Neuroendocrine tumor	8150-8153, 8155-8156, 8240-8241, 8244, 8249
Neuroendocrine carcinoma	8013, 8041, 8246
Solid pseudopapillary neoplasm	8452
Acinar cell carcinoma	8550
Other subtypes	(Other specified carcinoma)<br>8012, 8021-8022, 8030-8033, 8045-8046, 8050, 8070-8072, 8082, 8084, 8145, 8154, 8170, 8200, 8455, 8460, 8510, 8551, 8570, 8574-8576, 8803, 8971, 8980<br>(Unspecified carcinoma)<br>8000, 8010
