# Supplementary material for: Explainable artificial intelligence for personalized prognosis in pancreatic cancer: A nationwide study from Taiwan
Source: PLOS Digit Health. 2026 Mar 19;5(3):e0001296. doi: 10.1371/journal.pdig.0001296 (PMC13001956; doi:10.1371/journal.pdig.0001296)
Supplement: S2 Table — (PDF) [file pdig.0001296.s002.pdf]

S2 Table. Baseline characteristics and outcomes of pancreatic cancer patients.

Features	Male (n = 4,835)		Female (n = 4,029)	
	n	%	n	%
**Outcome (overall survival)**				
alive	798	16.50	855	21.22
dead	4,037	83.50	3,174	78.78
median survival time		9.02 months		10.56 months
**Age at diagnosis**				
20−39	99	2.05	135	3.35
40−49	391	8.09	257	6.38
50−59	1,074	22.21	697	17.30
60−69	1,612	33.34	1,297	32.19
70−79	1,084	22.42	1,020	25.32
80+	575	11.89	623	15.46
**BMI**				
<18.5	416	8.60	408	10.13
18.5−25	2,995	61.94	2,447	60.73
25−30	1,137	23.52	861	21.37
30+	205	4.24	205	5.09
unknown	82	1.70	108	2.68
**Subsite**				
head	2,259	46.72	1,870	46.41
body	797	16.48	663	16.46
tail	909	18.80	737	18.29
others	870	17.99	759	18.84
**Histological type**				
adenocarcinoma	3,771	77.99	3,058	75.90
neuroendocrine tumor	346	7.16	337	8.36
neuroendocrine carcinoma	93	1.92	52	1.29
solid papillary neoplasm	22	0.46	76	1.89
acinar cell carcinoma	26	0.54	13	0.32
others	577	11.93	493	12.24
**AJCC stage**				
I	437	9.04	473	11.74
II	1,033	21.37	874	21.69
III	698	14.44	659	16.36
IV	2,667	55.16	2,023	50.21
**T stage**				
T1	288	5.96	287	7.12
T2	1,104	22.83	976	24.22
T3	1,698	35.12	1,246	30.93
T4	1,581	32.70	1,388	34.45
missing	164	3.39	132	3.28
**N stage**				
N0	1,595	32.99	1,411	35.02
N1	2,390	49.43	1,875	46.54
N2	592	12.24	472	11.72
missing	258	5.34	271	6.73
**M stage**				
M0	2,183	45.15	2,015	50.01
M1	2,652	54.85	2,014	49.99

Features	Male (n = 4,835)		Female (n = 4,029)	
	n	%	n	%
**Grade**				
1	367	7.59	360	8.94
2	1,348	27.88	1,158	28.74
3	579	11.98	396	9.83
unknown	2,541	52.55	2,115	52.49
**Hospital level**				
medical center	3,772	78.01	3,093	76.77
others	1,063	21.99	936	23.23
**Urbanization level**				
1-2	353	7.30	251	6.23
3-5	1,570	32.47	1,315	32.64
6-7	2,912	60.23	2,463	61.13
**Years of smoking**				
0 (never)	2,524	52.20	3,778	93.77
1-10	618	12.78	63	1.56
10-20	265	5.48	40	0.99
20-30	310	6.41	33	0.82
30+	1,084	22.42	84	2.08
unknown	34	0.70	31	0.77
**Alcohol consumption**				
never	3,197	66.12	3,816	94.71
past	592	12.24	50	1.24
current	1,014	20.97	129	3.20
unknown	32	0.66	34	0.84
**Surgery**				
yes	1,624	33.59	1,431	35.52
no	3,211	66.41	2,598	64.48
**Chemotherapy**				
yes	3,449	71.33	2,701	67.04
no	1,386	28.67	1,328	32.96
**Radiotherapy**				
yes	571	11.81	463	11.49
no	4,264	88.19	3,566	88.51
**Targeted therapy**				
yes	266	5.50	207	5.14
no	4,569	94.50	3,822	94.86
**Hormonal therapy**				
yes	58	1.20	44	1.09
no	4,777	98.80	3,985	98.91
**Immunotherapy**				
yes	33	0.68	29	0.72
no	4,802	99.32	4,000	99.28
