# Supplementary material for: Explainable artificial intelligence for personalized prognosis in pancreatic cancer: A nationwide study from Taiwan
Source: PLOS Digit Health. 2026 Mar 19;5(3):e0001296. doi: 10.1371/journal.pdig.0001296 (PMC13001956; doi:10.1371/journal.pdig.0001296)
Supplement: S3 Table — (PDF) [file pdig.0001296.s003.pdf]

S3 Table. Hyperparameters and tuning ranges.

Models	Hyperparameters	Range	Optimal set
Cox proportional hazards models	L1 regularization penalty [1]	0.001λ – λ	
Oblique random survival forest (ORSF)	number of trees [2]	500	
	subsample ratio of predictors for splitting a node [3]	0.1 – 0.5	
	significance level of log-rank test for splitting a node [4]	0.001 – 0.1	
XGBoost	number of boosting rounds [5]	up to 5,000 steps with early stopping	1,165
	learning rate	0.001 – 0.1	0.0051
	maximum depth of a tree	4 – 8	5
	subsample ratio of predictors for splitting a node [3]	0.1 – 0.5	0.31
	subsample ratio of training data in each iteration [6]	0.5 – 1	0.81
	total amount of L1 and L2 regularization penalties [7]	10^{-5} – 10^{2}	8.4×10^{-5}
	mixing parameter denoting the proportion of L1 penalty [7]	0 – 1	0.63
Neural networks<br>• DeepSurv<br>• Cox-Time<br>• PC-Hazard	number of epochs [5]	up to 1,000 epochs with early stopping	
	learning rate	0.001 – 0.1	
	dropout ratio	0 – 0.5	
	number of hidden layers [8]	4 – 8	
	ratio of the first hidden layer width to the input layer width [8]	0.5 – 1	
	common ratio between layer widths [8]	0.5 – 1	

1 The `cv.grpsurv()` function in R `grpreg` package automatically computes λ, the smallest penalty value that shrinks all coefficients to zero, and then searches a sequence of penalty values down to 0.001×λ.

2 The prediction error of random forests typically stabilizes as the number of trees increases to an adequate level. In this study, we fix the number of trees at 500, a large enough and computationally feasible setting.

3 Drawing a subset of predictors for splitting a node can reduce correlation among trees and consequently decrease prediction variance. Commonly used sampling fraction is 1/3 or $1/\sqrt{p}$, where p is the number of predictors. A broader range (e.g. 0.1–0.5) can be considered for tuning.

4 The `aorsf` package in R uses the log-rank test as the stopping criterion for each node split during the tree-growing process, with the critical value set to 3.84 (corresponding to a significance level of 0.05). Thresholds of statistical significance that are more lenient or more stringent than 0.05 can be explored to allow for survival trees of varying depth.

5 Early stopping is triggered if the loss on the validation set does not improve over a specified number of steps.

6 Drawing a subset of training data for growing a tree in each boosting iteration can help prevent overfitting. The `XGBoost` online manual recommends setting this ratio greater than 0.5 for good results.

7 Regularization penalties are typically tuned on a log scale with search ranges spanning from values near zero (e.g. 10^{-5}) up to 10^{2} or even 10^{3}. `XGBoost` supports both L1 and L2 penalties. We reparametrize them into a total regularization magnitude, along with a mixing parameter ranging from 0 (L2 only) to 1 (L1 only) that controls the balance between both components.

8 We introduce 3 parameters to shape the monotonic network structures: k (number of hidden layers), q (ratio of the width in the first hidden layer to that in the input layer), and r (common ratio between the widths of consecutive layers). The number of nodes in the i-th hidden layer is given by

$$n_i = (n_0 \times q) \times r^{i-1} \quad \text{for } i = 1, \ldots, k$$

where n_0 is the number of nodes in the input layer. This allows for flexible architectures that can expand (r >1), contract (r <1), or remain uniform (r =1) in width across layers. Contracting and uniform settings tend to perform well empirically. As for q, a heuristic is to set the width of hidden layers to 2/3 of the sum of the input and output nodes. Accordingly, we use a broader range around this fraction for tuning.
