# Supplementary material for: Explainable artificial intelligence for personalized prognosis in pancreatic cancer: A nationwide study from Taiwan
Source: PLOS Digit Health. 2026 Mar 19;5(3):e0001296. doi: 10.1371/journal.pdig.0001296 (PMC13001956; doi:10.1371/journal.pdig.0001296)
Supplement: S4 Table — (PDF) [file pdig.0001296.s004.pdf]

S4 Table. Comparison with recent pancreatic cancer prognostic models.

Study	Data source	Sample size	Models	Key features	AUC
Yokoyama et al.<br>(2020; ref. 7)	Kagoshima University Hospital<br>Ulm University Hospital	191	For classification of high-risk group:<br>SVM, neural network, multinomial model<br><br>For survival prediction: Cox regression	mucin genes (MUC1, MUC2, MUC4), ASA score, preoperative chemotherapy, comorbidities, TNM stage	
Wang et al.<br>(2022; ref. 8)	The Cancer Genome Atlas (TCGA)<br>International Cancer Genome Consortium<br>ArrayExpress<br>Gene Expression Omnibus	1,280	random survival forest, lasso/ridge/elastic-net Cox, stepwise Cox, gradient boosting machine, survival-SVM, supervised principal components, partial least squares regression for Cox, CoxBoost	32 consensus prognostic genes selected by univariate Cox regression	1-year: 0.662-0.791<br>2-year: 0.683-0.865<br>3-year: 0.675-0.871<br><br>in the 9 testing cohorts
Chen et al.<br>(2024; ref. 9)	The Cancer Genome Atlas (TCGA)	185	CNN	differentially expressed genes selected by Lasso Cox	1-year: 0.789<br>3-year: 0.856<br>5-year: 0.838
Yao et al.<br>(2021; ref. 10)	Shengjing Hospital of China Medical University<br>Changhai Hospital<br>Memorial Sloan Kettering Cancer Center	1,209	3D ConvLSTM	CT image	1-year: 0.684<br>2-year: 0.689
Lee et al.<br>(2022; ref. 11)	Asan Medical Center, University of Ulsan	282	Ensemble model combining clinical data-based machine learning models<br>(logistic regression, random forest, gradient boosting machine, SVM, neural network)<br><br>and CT-based deep learning models<br>(3D ResNet-18, R(2+1)D-18,<br>3D ResNeXt-50, 3D DenseNet-121)	age, sex, BMI, ASA score, CA19-9, CEA, CT image	2-year: 0.76
Yao et al.<br>(2023; ref. 12)	Shengjing Hospital of China Medical University<br>Changhai Hospital<br>Tianjin Medical University Cancer Institute & Hospital<br>Guangdong Provincial People's Hospital<br>Sun Yat-sen University Cancer Center	1,516	3D ConvLSTM,<br>3D CNN	CT image	1-year: 0.756<br>2-year: 0.734<br>3-year: 0.703

Lin et al.<br>(2022; ref. 13)	Surveillance, Epidemiology, and End Results (SEER)	3,988	Cox regression,<br>random survival forest,<br>DeepSurv	prognostic factors selected by Lasso Cox: age, histologic type, AJCC stage, T stage, N stage, clinical grade, number of positive lymph nodes, rate of positive lymph nodes	1-year: 0.753<br>3-year: 0.744<br>5-year: 0.759
Keyl et al.<br>(2022; ref. 14)	University Hospital Essen<br>University Hospital Hamburg-Eppendorf	203	random survival forest	age, liver metastasis, C-reactive protein (CRP), neutrophil-to-lymphocyte ratio (NLR), CA19-9, total serum protein, KRAS mutation, CT image	
Teng et al.<br>(2024; ref. 15)	Surveillance, Epidemiology, and End Results (SEER)	20,064	random survival forest, lasso/ridge/elastic-net Cox, stepwise Cox, CoxBoost, partial least squares regression for Cox, supervised principal components, gradient boosting machine, survival-SVM	prognostic factors selected by uni- and multi-variate Cox regression: sex, race, age, marital status, household income, household location (urban/rural), tumor site, histology, grade, tumor size, AJCC stage, T stage, surgery type, number of regional lymph nodes removed, regional lymph nodes (negative/positive), radiotherapy, chemotherapy, bone/liver/lung metastasis	1-year: 0.796<br>3-year: 0.782<br>5-year: 0.761
Su et al.<br>(2024; ref. 16)	The Cancer Genome Atlas (TCGA)<br>GTeX<br>ImmPort<br>InnateDB	343	For classification of high-risk group: random forest, naive Bayes, KNN, decision tree, AdaBoost, Light GBM<br>For survival prediction: Cox regression	8 immune-related genes selected by Lasso Cox	1-year: 0.71<br>3-year: 0.76<br>5-year: 0.90
Our study	Taiwan Cancer Registry	8,864	Cox regression with group Lasso, machine learning models (XGBoost, oblique random survival forest), and deep learning models (DeepSurv, Cox-Time, PC-Hazard)	surgery, chemotherapy, histology, AJCC stage, T stage, N stage, M stage, grade, age, BMI, smoking duration	1-year: 0.847<br>3-year: 0.914<br>5-year: 0.936
