# Supplementary figures and images for: Explainable artificial intelligence for personalized prognosis in pancreatic cancer: A nationwide study from Taiwan
Source: PLOS Digit Health. 2026 Mar 19;5(3):e0001296. doi: 10.1371/journal.pdig.0001296 (PMC13001956; doi:10.1371/journal.pdig.0001296)

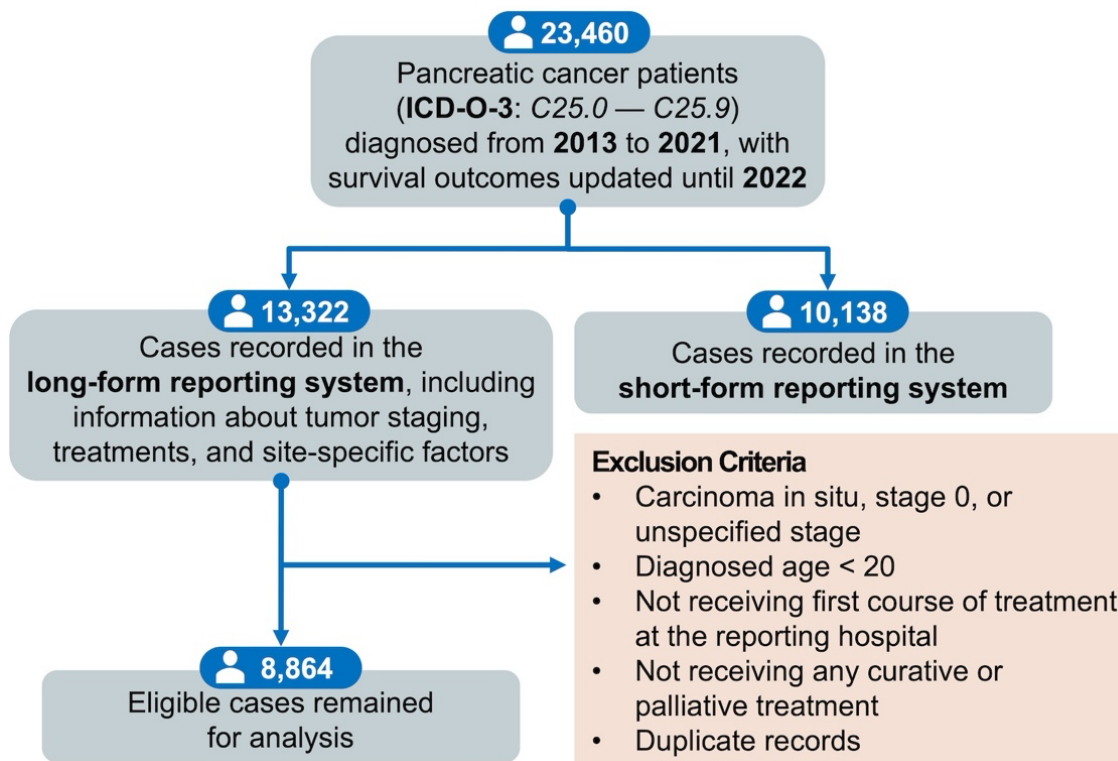

**S1 Fig.** Study flowchart.

Supplement: S1 Fig — (PDF) [file pdig.0001296.s005.pdf]

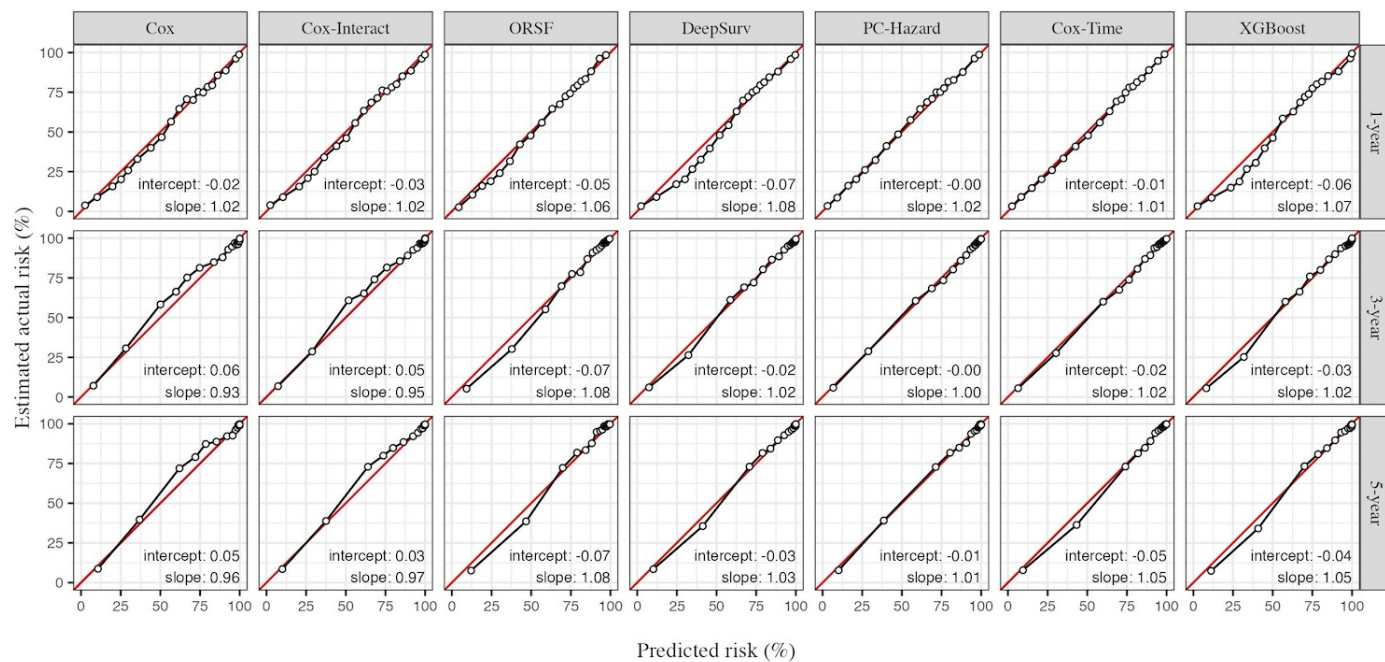

**S4 Fig.** Time-dependent calibration curves of the competing models.

Supplement: S4 Fig — (PDF) [file pdig.0001296.s008.pdf]
