# Supplementary material for: Explainable artificial intelligence for personalized prognosis in pancreatic cancer: A nationwide study from Taiwan
Source: PLOS Digit Health. 2026 Mar 19;5(3):e0001296. doi: 10.1371/journal.pdig.0001296 (PMC13001956; doi:10.1371/journal.pdig.0001296)
Supplement: S2 Fig — (PDF) [file pdig.0001296.s006.pdf]

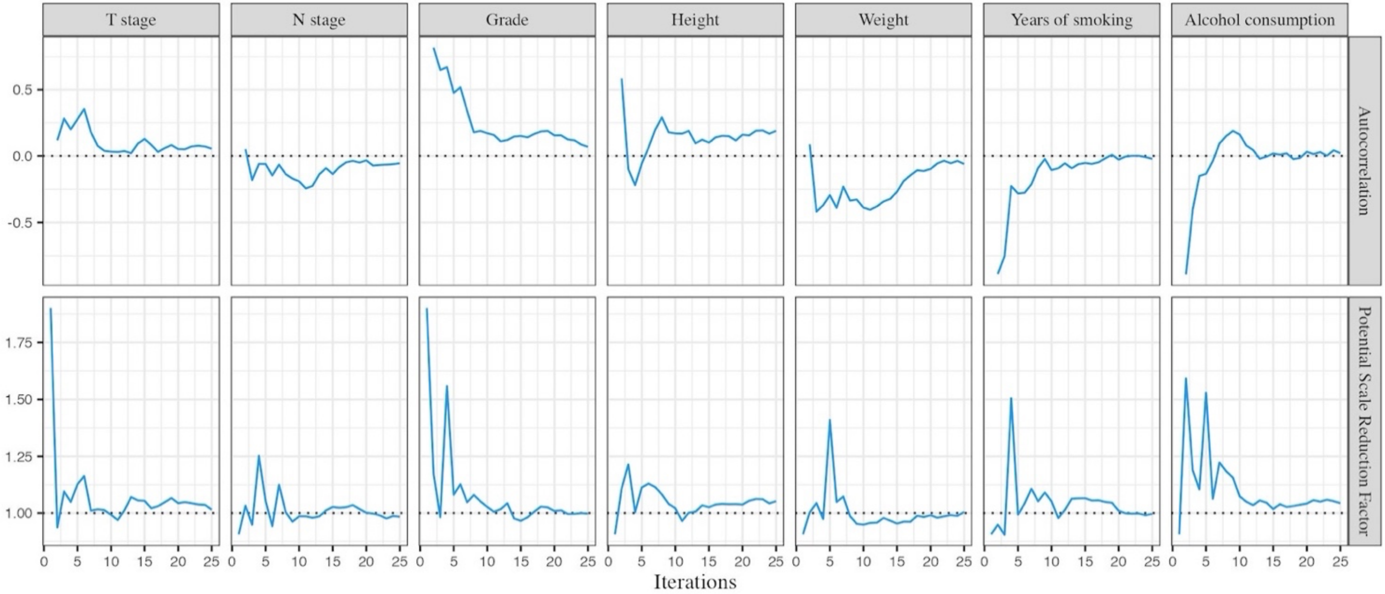

S2 Fig. MICE convergence diagnostics.

Convergence was assessed using autocorrelation and the potential scale reduction factor, with autocorrelations approaching zero and scale reduction factors approaching one indicating adequate convergence. Body mass index (BMI) was calculated after separately imputing height and weight.
