# Supplementary material for: Explainable artificial intelligence for personalized prognosis in pancreatic cancer: A nationwide study from Taiwan
Source: PLOS Digit Health. 2026 Mar 19;5(3):e0001296. doi: 10.1371/journal.pdig.0001296 (PMC13001956; doi:10.1371/journal.pdig.0001296)
Supplement: S3 Fig — (PDF) [file pdig.0001296.s007.pdf]

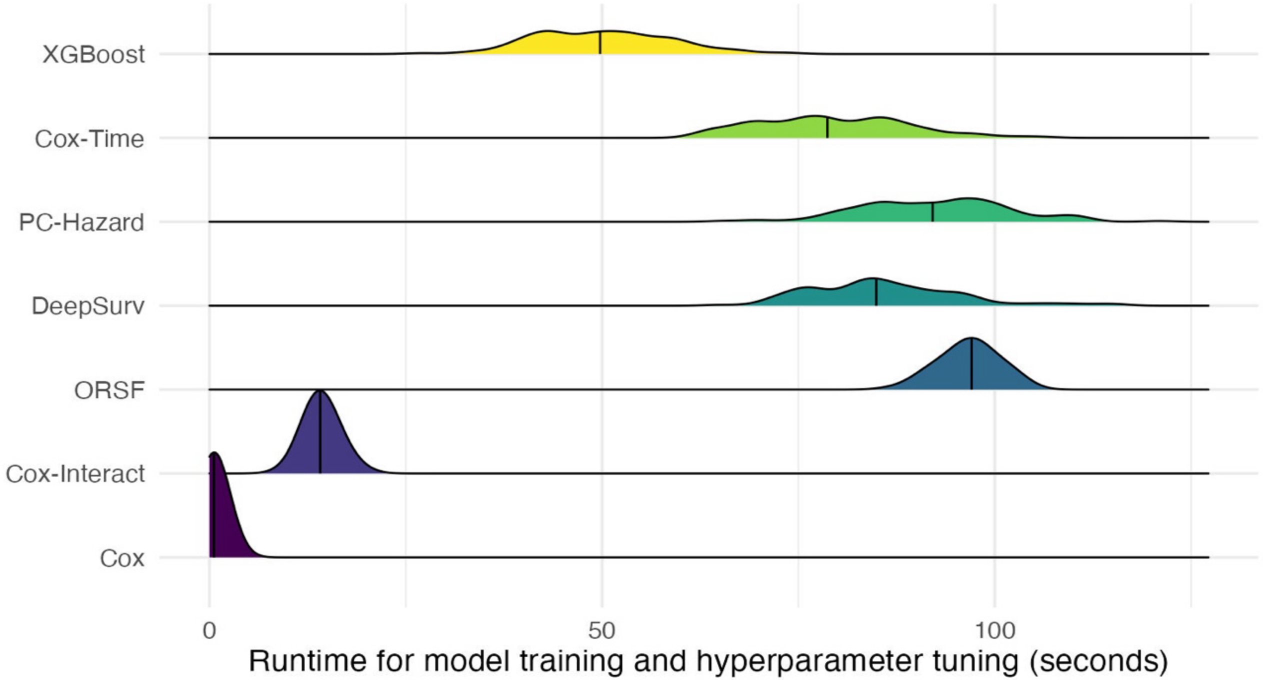

S3 Fig. Distribution of runtimes for model training and hyperparameter tuning.

The runtime is computed separately for every cross-validation split. The vertical line in each model's density plot represents the median time across all evaluated splits.
