# Supplementary material for: Explainable artificial intelligence for personalized prognosis in pancreatic cancer: A nationwide study from Taiwan
Source: PLOS Digit Health. 2026 Mar 19;5(3):e0001296. doi: 10.1371/journal.pdig.0001296 (PMC13001956; doi:10.1371/journal.pdig.0001296)
Supplement: S5 Fig — (PDF) [file pdig.0001296.s009.pdf]

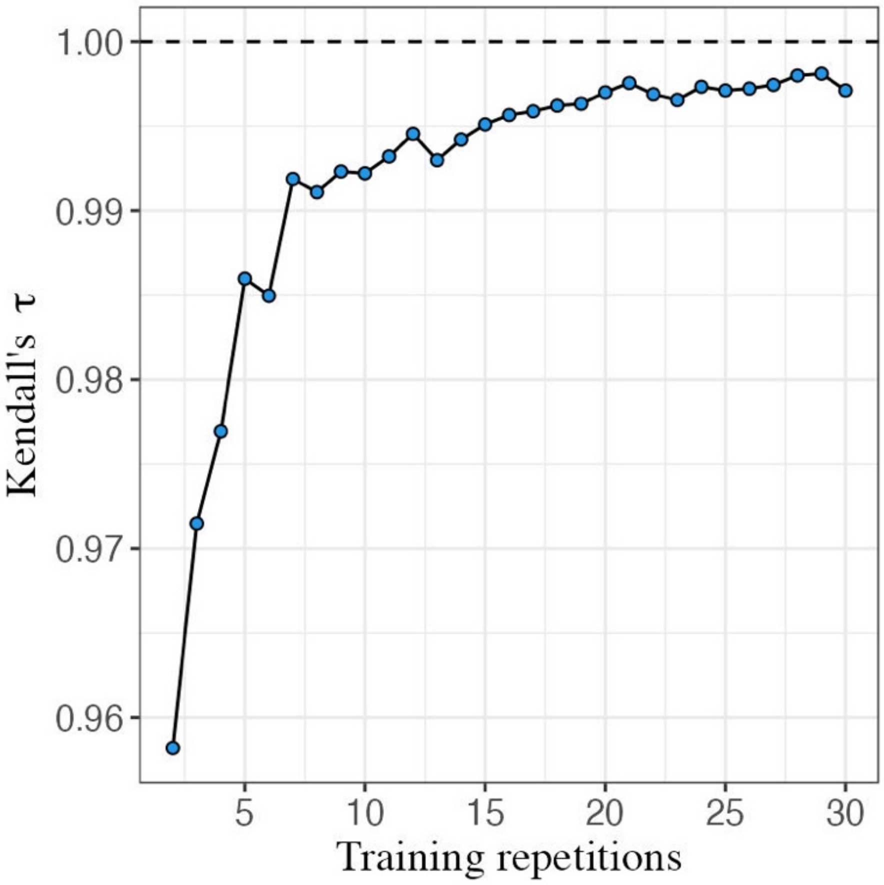

S5 Fig. Stability of the SHAP interaction importance rankings.

An XGBoost model with a set of fine-tuned hyperparameters (listed in S3 Table) was trained repeatedly, with the derived SHAP interaction values progressively averaged. The Kendall's τ correlation coefficients were calculated between successive rankings of the cumulative mean SHAP interaction values.
