# Supplementary material for: Explainable artificial intelligence for personalized prognosis in pancreatic cancer: A nationwide study from Taiwan
Source: PLOS Digit Health. 2026 Mar 19;5(3):e0001296. doi: 10.1371/journal.pdig.0001296 (PMC13001956; doi:10.1371/journal.pdig.0001296)
Supplement: S6 Fig — (PDF) [file pdig.0001296.s010.pdf]

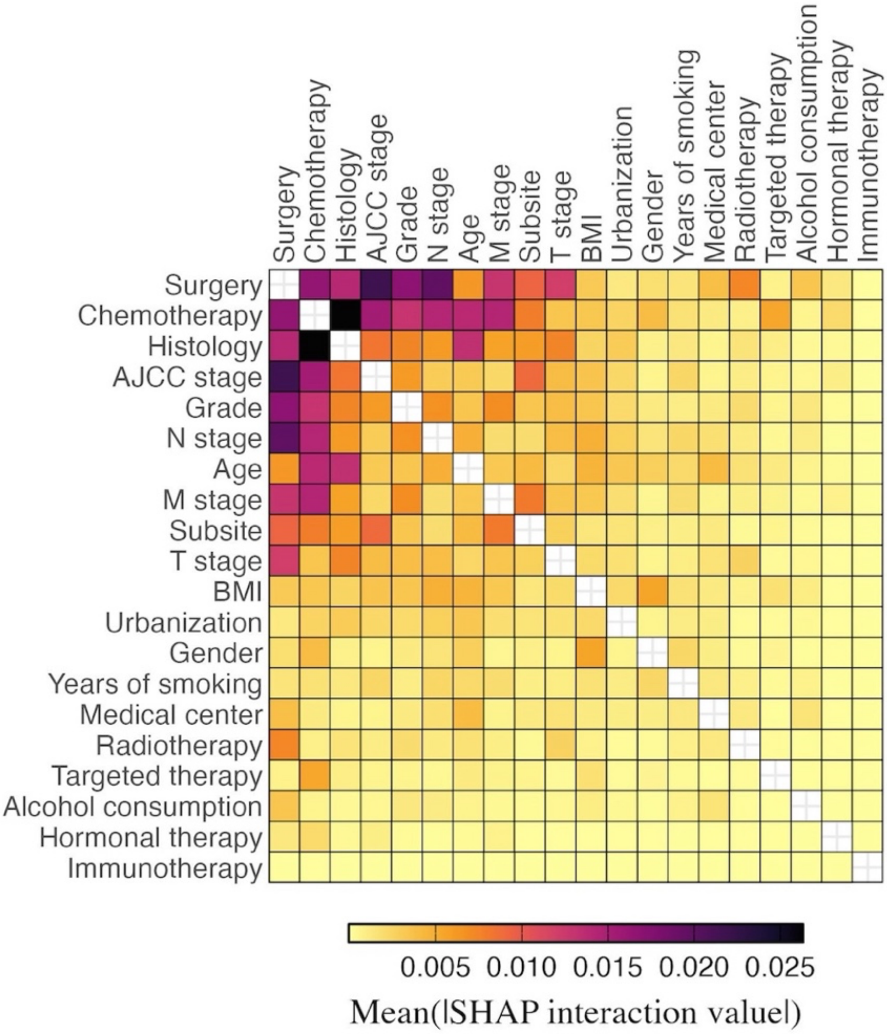

S6 Fig. Heatmap of SHAP interaction importance matrix.

SHAP interaction importance for each feature pair is measured by averaging the absolute SHAP interaction values across all patients. Features are arranged along both axes based on their mean interaction importance across all other features.
