# Supplementary material for: Explainable artificial intelligence for personalized prognosis in pancreatic cancer: A nationwide study from Taiwan
Source: PLOS Digit Health. 2026 Mar 19;5(3):e0001296. doi: 10.1371/journal.pdig.0001296 (PMC13001956; doi:10.1371/journal.pdig.0001296)
Supplement: S7 Fig — (PDF) [file pdig.0001296.s011.pdf]

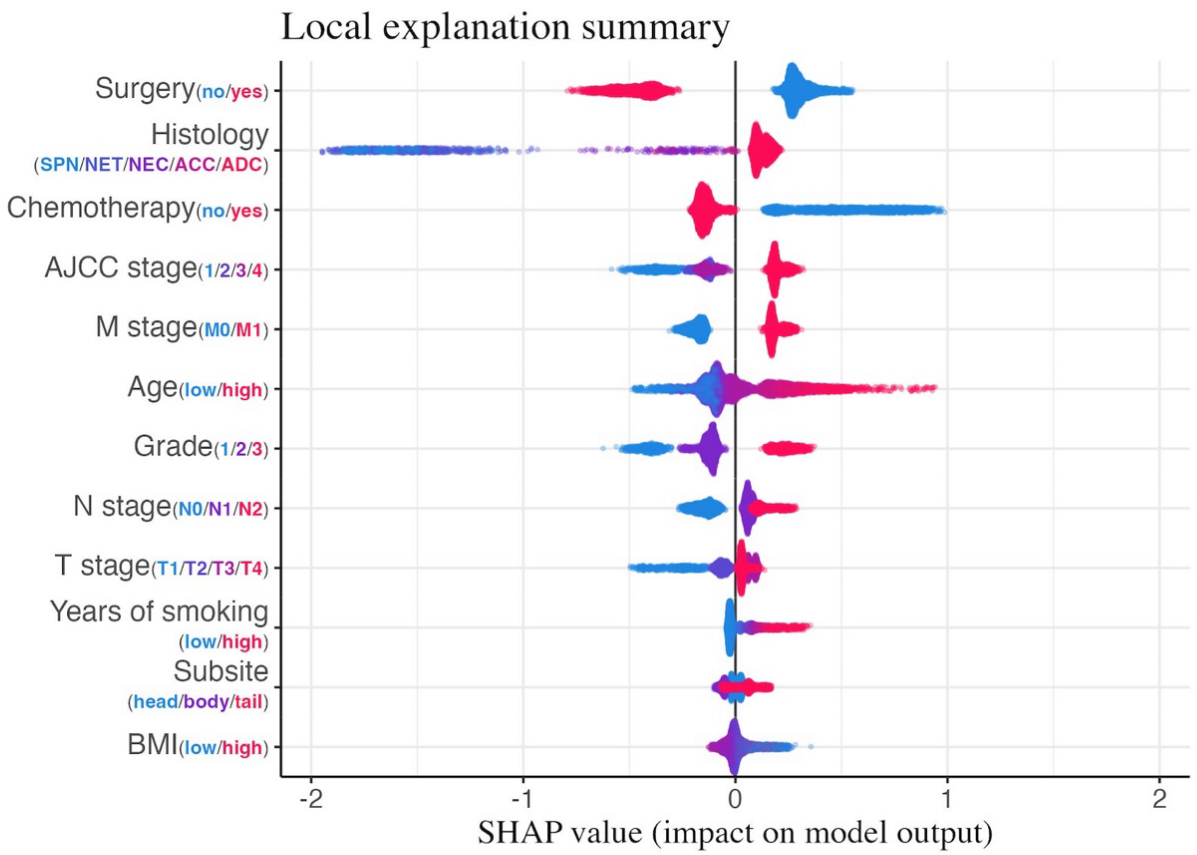

S7 Fig. SHAP summary plot for the 12 most important features.

Each point corresponds to an individual in the dataset, colored by its feature value. The SHAP summary plot is obtained by projecting the SHAP dependence plots from Figure 3 onto the y-axis. It succinctly visualizes the magnitude, distribution, and direction of each feature's effect on the predictions.
