# Supplementary material for: Explainable artificial intelligence for personalized prognosis in pancreatic cancer: A nationwide study from Taiwan
Source: PLOS Digit Health. 2026 Mar 19;5(3):e0001296. doi: 10.1371/journal.pdig.0001296 (PMC13001956; doi:10.1371/journal.pdig.0001296)
Supplement: S9 Fig — (PDF) [file pdig.0001296.s013.pdf]

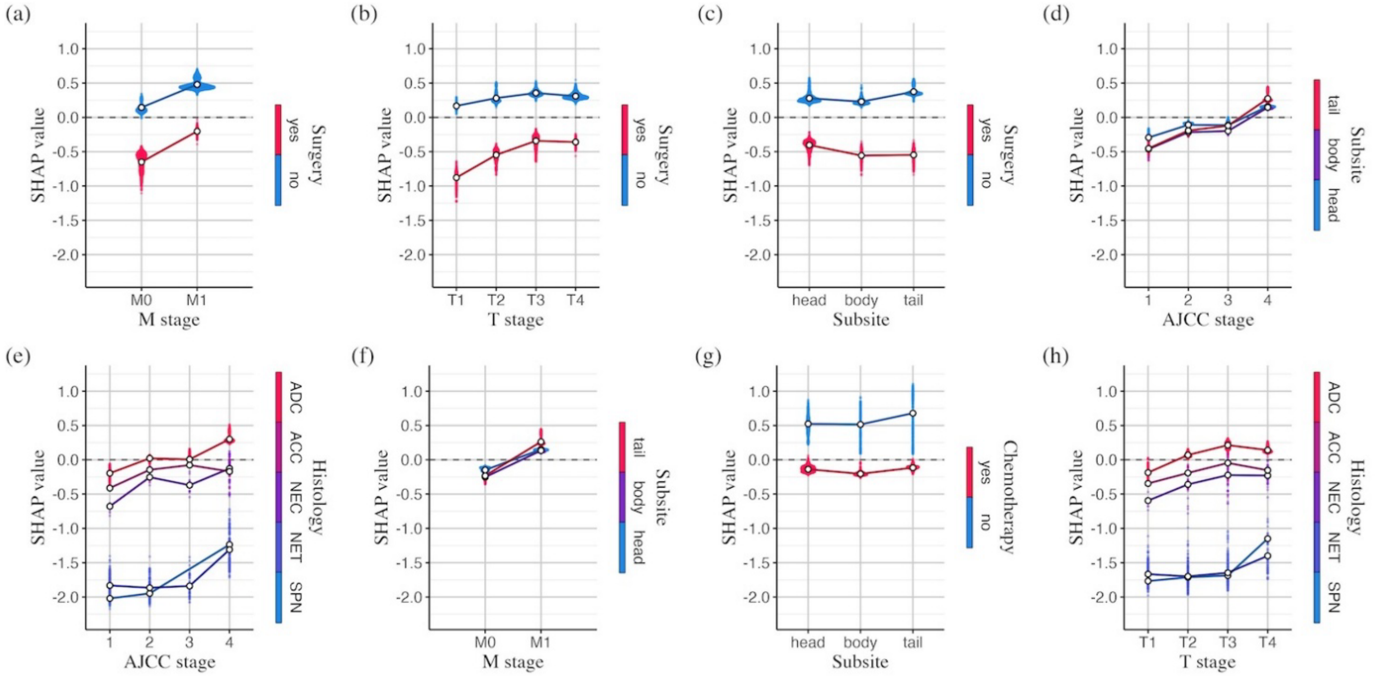

S9 Fig. SHAP dependence plots for feature pairs ranked 13–20.

(Dots for categorical variables: positions of the average SHAP values at each level; trend lines for continuous variables: fitted using locally estimated scatterplot smoothing; ADC: adenocarcinoma; NEC: neuroendocrine carcinoma; NET: neuroendocrine tumor; SPN: solid pseudopapillary neoplasm; ACC: acinar cell carcinoma)
