# Supplementary material for: Explainable artificial intelligence for personalized prognosis in pancreatic cancer: A nationwide study from Taiwan
Source: PLOS Digit Health. 2026 Mar 19;5(3):e0001296. doi: 10.1371/journal.pdig.0001296 (PMC13001956; doi:10.1371/journal.pdig.0001296)
Supplement: S10 Fig — (PDF) [file pdig.0001296.s014.pdf]

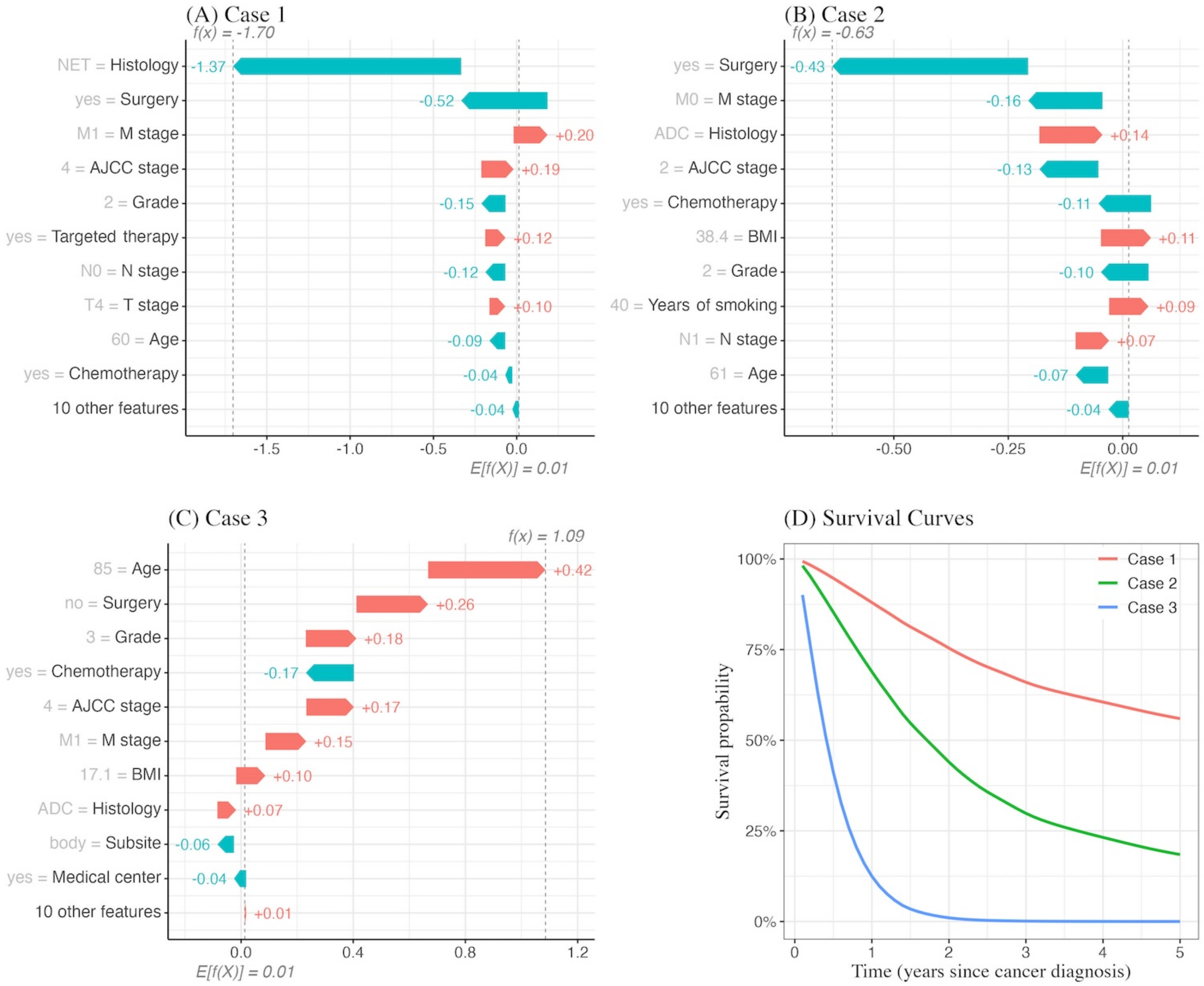

S10 Fig. Clinical vignettes of three example patients.

Panels (A)–(C) show the top 10 impactful features for each patient and their corresponding SHAP values. The length and color of each arrow bar indicate the magnitude and direction of that feature's contribution to the predicted risk, and the gray text before each feature name shows that patient's feature value. Panel (D) displays the predicted survival curves for each patient up to 5 years after cancer diagnosis. Case 1 was diagnosed with a neuroendocrine tumor, a subtype generally associated with more favorable survival; in this context, surgery was associated with substantial mortality reduction, whereas chemotherapy had only a limited effect even in stage IV disease. Case 2 was diagnosed with adenocarcinoma, the subtype with the highest mortality risk; long-term smoking and obesity further contributed to an adverse risk profile. However, early-stage detection without metastasis, together with both surgery and chemotherapy, corresponded to a more favorable predicted outcome. Case 3 was diagnosed with advanced adenocarcinoma with metastasis; older age and low BMI, potentially reflecting nutrient depletion or cachexia, contributed to the lowest predicted survival among the three cases.
