# Supplementary material for: Explainable artificial intelligence for personalized prognosis in pancreatic cancer: A nationwide study from Taiwan
Source: PLOS Digit Health. 2026 Mar 19;5(3):e0001296. doi: 10.1371/journal.pdig.0001296 (PMC13001956; doi:10.1371/journal.pdig.0001296)
Supplement: S11 Fig — (PDF) [file pdig.0001296.s015.pdf]

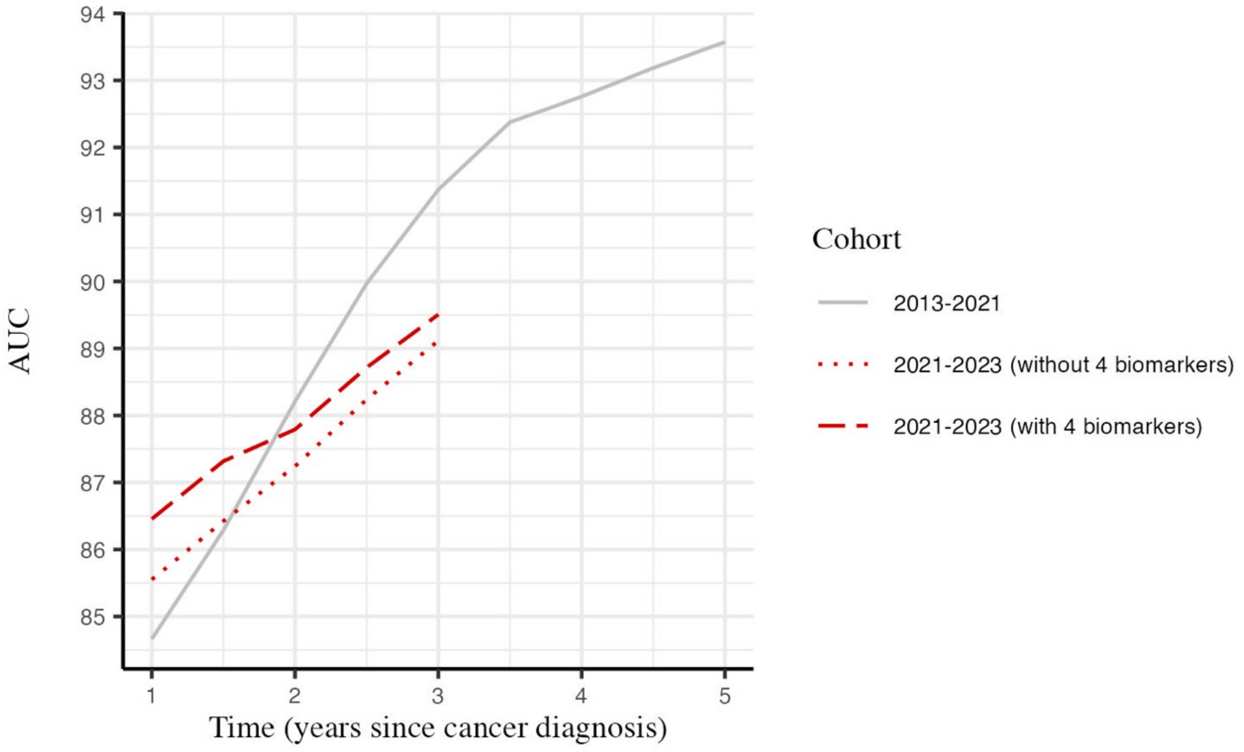

S11 Fig. Time-dependent AUCs from sensitivity analysis.

The sensitivity analysis compares XGBoost models with and without four additional biomarkers — diabetes history, HbA1c, CEA, and CA19-9.
