# Supplementary material for: Explainable artificial intelligence for personalized prognosis in pancreatic cancer: A nationwide study from Taiwan
Source: PLOS Digit Health. 2026 Mar 19;5(3):e0001296. doi: 10.1371/journal.pdig.0001296 (PMC13001956; doi:10.1371/journal.pdig.0001296)
Supplement: Attachment — Submitted filename: Peer Review Report - PDIG-D-25-00426.pdf [file pdig.0001296.s016.pdf]

PEER REVIEW REPORT

PLOS Digital Health
Manuscript ID: **PDIG-D-25-00426**

Title: Explainable Artificial Intelligence for Personalized Prognosis in Pancreatic Cancer: A Nationwide Study from Taiwan

Review Date: 10/7/2025

RECOMMENDATION: MINOR REVISIONS

Overall Assessment: This is a methodologically rigorous study with significant clinical relevance. The manuscript is technically sound and suitable for publication after addressing the issues outlined below. The authors have successfully developed an explainable AI model (XGBoost with SHAP) for pancreatic cancer prognosis using Taiwan's nationwide registry (8,864 patients), achieving strong predictive performance (C-index ~0.93) while maintaining interpretability.

PUBLICATION CRITERIA ASSESSMENT

Criterion	Assessment

Technically sound and methodologically rigorous?	**YES**
Data support conclusions?	**YES**
Statistical analysis appropriate?	**YES**
Conclusions appropriately stated and limited?	**YES**
Written in clear, standard English?	**YES***
Data availability statement adequate?	**PARTLY****

**Minor language corrections needed*

***Data appropriately restricted; code availability should be clarified*

KEY STRENGTHS

- **Large, high-quality dataset:** Nationwide population-based registry (8,864 patients, 2013-2021)
- **Rigorous methodology:** Nested cross-validation with 10 repeats; comprehensive model comparison (7 methods)
- **Strong performance:** XGBoost achieves C-index ~0.93, outperforming other approaches
- **Clinical relevance:** Identifies actionable treatment-histology interactions and non-linear relationships
- **Interpretability:** Effective use of SHAP values for explainable AI
- **Novel findings:** Chemotherapy most effective for adenocarcinoma (HR=0.460); surgery for neuroendocrine tumors (HR=0.378); age threshold effect at 65 years

MAJOR ISSUES REQUIRING REVISION

ISSUE 1: Treatment Selection Bias and Causal Language

Priority: HIGH | Lines: 195-200, 221-236, 261-275

Problem: The manuscript uses causal language to describe treatment effects from an observational study. Patients receiving surgery/chemotherapy likely differ systematically from those who don't on unmeasured confounders (performance status, frailty, comorbidities). The findings represent **prognostic associations**, not **causal effects**.

Current problematic statements:

- Line 195-200: "surgery and chemotherapy substantially *reduce* mortality risk"
- Line 222: "Chemotherapy *provides* the most substantial reduction"
- Line 224: "surgery is *especially beneficial*"
- Lines 261-275: Discussion interprets findings as informing treatment decisions

REQUIRED ACTIONS:

1. Add to Limitations section (after line 276):

"This observational study cannot establish causal treatment effects due to potential confounding by indication. Patients selected for surgery or chemotherapy differ systematically from those who aren't based on unmeasured factors such as performance status, functional capacity, frailty, and disease progression rate—all of which independently affect survival. The identified treatment effects represent **prognostic**

associations in our model rather than causal estimates. These associations should be validated in randomized trials or studies with robust confounding control (e.g., propensity score methods, instrumental variables) before directly informing treatment allocation decisions."

2. Revise Results section (Lines 195-200):

Change from: "surgery and chemotherapy substantially reduce mortality risk"
Change to: "surgery and chemotherapy are associated with lower mortality risk in our prognostic model"

3. Revise Results section (Lines 222-236):

- Line 222: "Chemotherapy is *associated with* the most substantial reduction" (not "provides")
- Line 224: "surgery is *associated with particularly low mortality risk* for neuroendocrine tumors" (not "especially beneficial")
- Throughout: Replace "effectiveness," "benefit," "effective" with "associated with lower/higher mortality risk"

4. Add to Discussion (after line 268):

"While these associations suggest differential treatment effectiveness across patient subgroups, they must be interpreted cautiously given the observational study design. Validation in randomized controlled trials or adequately controlled observational studies is essential before using these findings to guide treatment selection. Our model is **prognostic** (predicting outcomes given treatments received) rather than **prescriptive** (recommending optimal treatment)."

Recent Context: Multiple 2024 studies using similar XGBoost+SHAP approaches explicitly distinguish prognostic from causal effects. A 2024 Scientific Reports study states: "ML models identify prognostic associations but cannot replace randomized evidence for treatment decisions."

ISSUE 2: Clinical Implementation and Utility

Priority: HIGH | Section: Discussion

Problem: The manuscript demonstrates strong statistical performance but doesn't adequately articulate practical clinical utility. Recent literature emphasizes AI models must show clear clinical value beyond performance metrics.

Current gaps:

- No concrete examples of clinical use
- No discussion of implementation feasibility
- Limited connection to treatment guidelines
- No decision support demonstration

REQUIRED ACTIONS:

1. Add new subsection to Discussion (after line 275):

Clinical Applications and Implementation Considerations

Our explainable AI model could support clinical decision-making in several ways:

Risk Stratification: The model could identify high-risk patients requiring intensive monitoring, early palliative care discussions, or aggressive multimodal therapy. For example, our findings suggest that patients with adenocarcinoma aged >65 years with advanced stage disease represent a particularly high-risk group where chemotherapy benefit is most pronounced.

Treatment Pattern Recognition: The model reveals important associations that warrant clinical attention:

- Adenocarcinoma patients show the strongest association between chemotherapy and lower mortality (HR=0.460)
- Neuroendocrine tumor patients show particularly strong association with surgery (HR=0.378)
- The chemotherapy-mortality association strengthens with age, peaking around 65 years
- Surgery shows strongest association with survival in early-stage disease

Personalized Counseling: SHAP values provide patient-specific risk contributions, enabling transparent discussions about prognosis. Clinicians could explain which factors most influence an individual patient's predicted outcome.

Implementation Requirements: Before clinical deployment, the model requires: (a) prospective validation in independent cohorts, (b) integration with electronic health record systems, (c) user-friendly interface development, (d) clinician training programs, and (e) monitoring systems for model performance drift over time.

2. Add to Supplementary Materials:

Clinical vignettes showing 2-3 example patients with different characteristics, demonstrating:

- Input features
- Predicted survival curves (1, 3, 5 years)
- SHAP value explanations
- Clinical interpretation

Justification: Recent reviews note that 90% of clinical AI tools fail adoption due to unclear clinical utility. MIT's PRISM pancreatic cancer model (2024) includes explicit implementation pathway discussion.

ISSUE 3: Code Availability and Reproducibility

Priority: HIGH | Section: Methods/Data Availability

Problem: The manuscript doesn't mention code availability, limiting reproducibility. PLOS policies increasingly emphasize code sharing for computational studies.

REQUIRED ACTION:

Add to end of Methods section:

Code Availability

All analysis code will be made publicly available upon acceptance at [GitHub/Zenodo repository URL]. The repository includes:

- Data preprocessing and cleaning scripts
- Multiple imputation implementation (MICE with random forests)
- Model training code for all seven methods (Cox, Cox-Interact, XGBoost, ORSF, DeepSurv, Cox-Time, PC-Hazard)
- Nested cross-validation framework
- SHAP value calculation and visualization code

- Time-dependent metric computation
- Synthetic data example with similar structure for testing
- Environment specifications (requirements.txt, Python version, package versions)

Due to privacy restrictions under Taiwan's Personal Information Protection Act, actual patient data cannot be shared. However, synthetic data with identical structure and similar statistical properties is provided to enable reproducibility verification.

Alternative add to Data Availability Statement:

"Analysis code will be deposited in a public repository (GitHub/Zenodo) with permanent identifier upon manuscript acceptance."

Justification: Code sharing is essential for reproducibility and is increasingly required by journals. Synthetic data approach allows methods verification while protecting privacy.

ISSUE 4: Missing Biomarkers Impact

Priority: MEDIUM | Lines: 276-281

Problem: Key prognostic biomarkers (CA19-9, CEA, diabetes, HbA1c) were only available from 2021 onward. Current acknowledgment (lines 276-281) is insufficient—the impact should be quantified.

REQUIRED ACTIONS:

1. Conduct sensitivity analysis (if 2021 data accessible):

- Train models on 2021 subset WITH biomarkers
- Compare C-index with vs without biomarkers
- Report in main text or supplementary materials

2. Revise Limitations section (lines 276-281):

"This study has several limitations. First, key prognostic biomarkers (CA19-9, CEA) and comorbidity indicators (diabetes history, HbA1c) were only available from 2021 onward and thus could not be included in our primary analysis. [*If sensitivity analysis conducted:* In sensitivity analysis using the 2021 cohort (n=XXX), including these biomarkers improved model C-index from X.XX to X.XX, suggesting potential for performance gains.] However, our model without these biomarkers achieved strong discrimination (C-index=0.93), and the identified patterns—particularly treatment-histology interactions and non-linear age effects—likely remain valid. Future studies incorporating these biomarkers alongside genomic features (e.g., KRAS, TP53 mutations) may further enhance prediction and reveal additional interaction patterns."

Recent Context: A 2024 Informatica study showed CA19-9 addition improved pancreatic cancer prediction from 92% to 95%. Multiple studies identify diabetes as doubling pancreatic cancer mortality risk.

MINOR ISSUES

Multiple Imputation Reporting (Lines 138-141)

Add:

- Supplementary table showing proportion of missing data for each variable
- Statement on MICE convergence diagnostics
- Report whether SHAP value rankings were consistent across five imputed datasets

Example: "Missing data ranged from X% (variable) to Y% (variable). MICE algorithm convergence was confirmed by trace plot inspection. Feature importance rankings showed high consistency across imputed datasets (mean Kendall's τ = 0.XX)."

Calibration Assessment

Add:

- Calibration curves at 1, 3, and 5 years post-diagnosis
- Report calibration slope and intercept (perfect calibration: slope=1, intercept=0)
- Include in supplementary materials

Rationale: Study reports discrimination (AUC, C-index) but not calibration. For clinical use, knowing whether predicted probabilities match observed outcomes is essential.

Comparison with Prior Models (Lines 238-253)

Add to Discussion:

Brief quantitative comparison with recent pancreatic cancer prognostic models:

- Yokoyama et al. (2020): ML on clinical+genomic data
- Wang et al. (2022): Comprehensive ML framework
- Lin et al. (2022): Random survival forest on SEER data
- Teng et al. (2024): Interpretable ML on population data

Include comparison table in supplementary materials with: Study | Year | N | Method | C-index | Key Features

Effect Heterogeneity Quantification (Lines 221-236, Figure 4)

Add:

- Bootstrap confidence intervals for key hazard ratios derived from SHAP values
- Example: "Chemotherapy for adenocarcinoma (HR=0.460, 95% CI: X.XX-X.XX)"
- Add to Methods: "Uncertainty in SHAP-derived hazard ratios quantified using 1,000 bootstrap iterations"

External Validation Discussion (Lines 276-281)

Add to Limitations/Future Directions:

"External validation in independent cohorts is essential. Priorities include: (1) geographic validation in Western populations, (2) temporal validation on recent data (2022-2024), (3) prospective validation in clinical settings, and (4) cross-ethnic validation. Our reliance on routinely collected clinical data enhances implementation potential, but population-specific calibration may be needed."

Figure 1 Performance Plateau (Lines 172-179)

Add brief explanation after line 179:

"The performance plateau after year 3 reflects: (1) most deaths occurring within first 3 years, providing richer training signal, and (2) increasing censoring reducing events for evaluation."

Mechanistic Context for Non-linear Relationships (Lines 254-260)

Add brief biological explanations:

- **BMI U-shape:** Low BMI reflects cachexia/nutritional depletion; high BMI indicates metabolic dysfunction, inflammation
- **Age threshold at 65:** Reflects declining physiological reserve, increased comorbidity burden, age-dependent tumor biology
- **Smoking plateau:** May reflect survivor bias and competing mortality risks

Hyperparameter Transparency (Lines 145-147, Supp Table S2)

Add to supplementary materials:

- Rationale for hyperparameter search ranges
- Final selected hyperparameters for best model (XGBoost)
- Computational time information

Language and Formatting Corrections

Minor corrections needed:

- Line 67-68: Fix "7-968" → Should be "7-9" (reference formatting error)
- Lines 84, 107, 118: Add spaces before superscript citations
- Line 99-100: Fix "Cancer(AJCC)" → "Cancer (AJCC)"
- Lines 85-93: Break long sentence into 2-3 shorter sentences
- Line 53: Change "highly developed countries" → "high-income countries" (standard WHO terminology)
- Line 210: Clarify "0.69% per year" (percentage or percentage points?)
- Ensure consistent abbreviation formatting throughout (space before parentheses)
