# Supplementary material for: Explainable artificial intelligence for personalized prognosis in pancreatic cancer: A nationwide study from Taiwan
Source: PLOS Digit Health. 2026 Mar 19;5(3):e0001296. doi: 10.1371/journal.pdig.0001296 (PMC13001956; doi:10.1371/journal.pdig.0001296)
Supplement: Attachment — Submitted filename: Response to Reviewers Comments.docx [file pdig.0001296.s018.docx]

**Response to Reviewers’ Comments**

**Reviewer #1**

This manuscript meets the publication criteria of PLOS Digital Health. It presents a well-executed, methodologically sound, and ethically approved study applying explainable AI models to a nationwide dataset to improve pancreatic cancer prognosis. The conclusions are clearly supported by the data and analyses.

**Response**:

Thank you for your positive and constructive comments.

But there are minor issues:

- Model calibration: The manuscript focuses on discrimination (AUC) but does not discuss model calibration (e.g., observed vs. predicted survival curves). Including this would further strengthen confidence in clinical applicability.
- Reproducibility: The authors should release the code to enable verification.
- Could the authors elaborate on why XGBoost was selected as the final model, given that multiple modeling approaches were tested?

**Response**:

Thank you for highlighting these important issues.

1. We added time-dependent calibration curves to assess agreement between predicted risks and observed outcomes, as described in the Results section:

“The calibration curves of all competing models at 1, 3, and 5 years after cancer diagnosis are shown in Supplementary Figure S4, and the corresponding calibration slopes and intercepts indicate that predicted survival closely matches observed outcomes across models”.

2. We released the analysis code and updated the Data Availability Statement:

“The analysis code and a synthetic dataset with similar structure and statistical properties to the real data will be deposited in a public GitHub repository (https://github.com/darentsai/SurvPancreas), with permanent identifier upon manuscript acceptance”.

The versions of the software and required packages are documented in the website.

3. We elaborated on XGBoost’s performance in the Results section:

- “XGBoost had the highest sensitivity before year 3 and the highest specificity thereafter.”
- “The time-dependent AUC and scaled Brier score curves show widening performance differences over time, with XGBoost consistently achieving the highest values across the full time horizon.”

These results support the choice of XGBoost as the final model.

**Reviewer #2**

This is a valuable and timely study, and I appreciate the use of a large national dataset together with explainable AI methods. The manuscript is clearly written and the clinical insights are compelling.

**Response**:

Thank you for your positive and constructive comments.

That said, two major issues need attention before the results can be considered robust. First, the way treatments (surgery, chemotherapy, etc.) are coded as baseline variables introduces immortal-time bias and likely overstates their benefit; these should be modeled as time-dependent or handled with landmark analyses. Second, missing data were imputed before cross-validation, which risks information leakage from the validation folds and could make model performance appear better than it really is; imputation should be nested within each training fold. Beyond these, I encourage the authors to add calibration plots, clarify the XGBoost survival objective and SHAP interpretation, and, if possible, perform temporal validation. Addressing these points will greatly strengthen the manuscript and ensure its conclusions are reliable. Otherwise, great contribution.

**Response**:

Thank you for highlighting these important issues.

1. We have acknowledged the reviewer’s concern regarding coding treatments as baseline variables as an important limitation in the Discussion:

“Second, treatments were coded as baseline “ever/never” variables, which may introduce immortal-time bias because patients must survive long enough after diagnosis to receive surgery or chemotherapy; this can overstate apparent associations by attributing pre-treatment survival time to the treated group. Future analyses should model treatments as time-dependent covariates or use landmark analyses to better align exposure definition with clinical timing.”

2. We agree that imputation should be nested within each training fold to avoid information leakage from the validation folds, which can lead to optimistic performance estimates. Accordingly, we added the following limitation to the Discussion:

“Methodologically, imputing before cross-validation may leak information via validation-fold covariate distributions and inflate performance. Although follow-up time and event status were excluded to prevent outcome-driven leakage, future analyses should nest imputation within training folds; for deployment, imputation models should be trained on development data and applied to new patients.”

3. We added time-dependent calibration curves to assess agreement between predicted risks and observed outcomes, as described in the Results section:

“The calibration curves of all competing models at 1, 3, and 5 years after cancer diagnosis are shown in Supplementary Figure S4, and the corresponding calibration slopes and intercepts indicate that predicted survival closely matches observed outcomes across models.”

4. We clarified the XGBoost’s survival objective in the Methods section:

“We specified the Cox partial log-likelihood as the objective function in XGBoost to predict log hazard ratios (HRs) for mortality, and then estimated the baseline hazard using the Breslow method to derive survival probabilities.”

5. We performed temporal validation using recent data, and discussed the need for additional external validation in the Discussion section:

“Finally, external validation in independent cohorts remains essential: temporal validation in 2022–2023 yielded 1-year and 2-year AUCs of 0.858 and 0.878, respectively, but geographic and cross-ethnic validation is still needed to establish generalizability across populations and healthcare settings.”

**Reviewer #3**

1. Generalizability and External Validation

Although the study benefits from the use of a large and representative nationwide cancer registry in Taiwan, the model has only been validated internally through nested cross-validation. Without external validation on an independent cohort, questions remain regarding how well the findings generalize to other populations, particularly given differences in histological subtype distributions and BMI ranges.

**Response**:

Thank you for highlighting this important issue. We performed temporal validation using recent data, and discussed the need for additional external validation in the Discussion section:

“Finally, external validation in independent cohorts remains essential: temporal validation in 2022–2023 yielded 1-year and 2-year AUCs of 0.858 and 0.878, respectively, but geographic and cross-ethnic validation is still needed to establish generalizability across populations and healthcare settings.”

2. Deep Learning vs. Tree-based Models

The results show that XGBoost outperforms deep learning models, consistent with previous reports on tabular data. However, the deep learning architectures applied were relatively basic. This limitation should be more clearly acknowledged, as the weaker performance may reflect architectural choices and data modality rather than an inherent disadvantage of deep learning.

**Response**:

Thank you for your suggestion. We revised our comments regarding the performance differences between the two models in the Discussion section:

“Although our findings in real-world pancreatic cancer survival data are consistent with this pattern, it may partly reflect the relatively simple neural network architectures used in our study”.

3. Clinical Utility and Interpretability

The application of SHAP to explain the model is a clear strength. Still, the interpretation remains largely visual, and many interactions are not translated into clinically meaningful insights. For instance, patterns involving BMI and age or smoking duration and chemotherapy could be more fully discussed in the context of treatment relevance.

**Response**:

Thank you for your suggestion. We added clinical explanations to the revealed nonlinear effects of age, smoking, and BMI on pancreatic cancer survival in the Discussion section:

- “The increase in mortality risk accelerates after age 65, which may relate to declining physiological reserve, accumulating comorbidities, and age-dependent tumor biology.”
- “In our analyses, mortality risk increases with longer smoking duration but appears to plateau between 10 and 30 years, which may reflect survivor bias and competing risks.”
- “Low BMI may reflect nutrient depletion or cachexia, whereas high BMI is indicative of metabolic dysfunction and chronic inflammation.”

4. Censoring in Survival Analysis

The manuscript applies inverse probability of censoring weighting but does not report the proportion of censored observations. This information is essential in survival analysis to evaluate the reliability of the models and should be described explicitly.

**Response**:

Thank you for your suggestion. We added the baseline characteristics of the predictors and outcomes in Supplementary Table S2. The proportion of censored observations are reported in the table.

Overall Assessment

This is a well-designed study with a strong dataset and rigorous methodology, and it makes a valuable contribution to the field of explainable AI in oncology. With clearer discussion of generalizability, model limitations, interpretability, and censoring, the manuscript would be substantially strengthened. These clarifications do not require major reanalysis, and the study is suitable for publication after minor revisions.

**Response**:

Thank you for your positive and constructive comments.

**Reviewer #4**

*Summary*

This manuscript presents a methodologically rigorous study using explainable AI to predict pancreatic cancer survival from Taiwan's nationwide cancer registry (8,864 cases, 2013-2021). The authors compare multiple modeling approaches and demonstrate that XGBoost outperforms other methods. The use of SHAP values to identify prognostic factors, interactions, and non-linear relationships is innovative and clinically relevant. The manuscript is well-written and makes important contributions to precision oncology. I recommend Minor Revisions to address the issues below.

*Major Strengths*

1. Large, high-quality nationwide population-based dataset with excellent data completeness

2. Rigorous methodology with nested cross-validation and appropriate time-dependent metrics

3. Comprehensive comparison of regression, machine learning, and deep learning approaches

4. Novel application of SHAP to reveal clinically meaningful interactions (treatment × histology)

5. Identification of non-linear relationships (age, BMI, smoking) often missed by traditional models

6. Clear documentation of ethical approval and data handling procedures

**Response**:

Thank you for your positive and constructive comments.

*Major Issues Requiring Revision*

1. Treatment Selection Bias and Causal Interpretation (High Priority)

Issue: The study identifies important treatment effects and interactions, but patients receiving surgery or chemotherapy likely differ systematically from those who don't (performance status, comorbidities, disease severity). The manuscript should more clearly distinguish between prognostic associations and causal treatment effects.

Specific Recommendations:

- Add discussion of confounding by indication in the limitations section
- Clarify throughout (especially in Results lines 195-200 and Discussion lines 261-275) that identified treatment effects represent associations in a prognostic model rather than causal effects
- Acknowledge unmeasured confounders (performance status, frailty, disease progression rate) that may influence both treatment selection and outcomes
- Consider adding: "These findings suggest differential treatment effectiveness across patient subgroups but should be validated in randomized trials or studies with robust confounding control before informing treatment decisions"

**Response**:

Thank you for highlighting this important issue. We revised causal language regarding treatment effects throughout the manuscript to reflect prognostic associations and addressed this concern as a limitation in the Discussion:

“One key limitation is the observational design, as treatment–outcome associations may be influenced by residual confounding, confounding by indication, and treatment selection bias (e.g., unmeasured differences in performance status, frailty, or disease progression between treated and untreated patients). Accordingly, the observed effects of surgery and chemotherapy represent prognostic associations rather than causal estimates and should be interpreted with caution; these associations should be validated in randomized trials or rigorously controlled observational studies before being used to inform treatment allocation. In addition, because the model is prognostic rather than prescriptive, it does not generate treatment recommendations, and we did not assess concordance between model outputs and clinical treatment decisions.”

2. Clinical Implementation and Utility (High Priority)

Issue: While the study demonstrates strong predictive performance and identifies important patterns, the practical clinical utility needs clearer articulation.

Specific Recommendations:

- Provide concrete clinical examples (e.g., "For a 70-year-old patient with stage IIA adenocarcinoma, our model predicts...")
- Discuss feasibility of implementing this as a clinical decision support tool
- Address how findings might inform treatment guidelines or shared decision-making
- Consider adding a supplementary clinical vignette demonstrating personalized risk prediction for 2-3 prototypical patients

**Response**:

Thank you for highlighting this important issue. We added two paragraphs to the Discussion section to describe potential clinical applications and implementation considerations, and we also included clinical vignettes for three example patients:

“Our explainable AI model could support clinical decision-making in several ways. First, it may assist risk stratification by identifying patients at elevated risk who could benefit from closer monitoring, earlier integration of palliative care, or consideration of more intensive treatment options. The treatment-related interactions revealed by the model also help characterize how prognosis varies across clinical subgroups. For example, our findings suggest that older patients with advanced-stage adenocarcinoma constitute a particularly high-risk group in whom the mortality reduction associated with chemotherapy appears most pronounced, a pattern that may warrant further investigation in dedicated clinical or observational studies”.

“Second, patient-level SHAP values enable transparent, individualized risk communication: clinicians can show which factors contribute most to a specific patient’s predicted risk and how changes in key variables (e.g., smoking duration, BMI) might influence prognosis. Supplementary Figure S10 illustrates clinical vignettes for three example patients with distinct characteristics, displaying their input features, corresponding SHAP values, predicted survival curves, and possible clinical interpretation. Finally, several implementation issues would need to be addressed before clinical deployment, including integration with electronic health record systems, development of user-friendly interfaces, clinician training on model interpretation, and ongoing surveillance for performance drift over time. Together, these elements outline a potential pathway for translating our model into a practical clinical decision-support tool, while recognizing that its role should complement, rather than replace, clinical judgment and guideline-based care”.

3. Missing Biomarkers Impact Assessment (Medium Priority)

Issue: Important prognostic factors (CEA, CA19-9, HbA1c, diabetes history) were only available from 2021 onward, but the impact of their absence isn't adequately quantified.

Specific Recommendations:

- Conduct sensitivity analysis using 2021 data subset that includes these biomarkers
- Report model performance with vs. without these additional features
- Estimate potential performance improvement if these variables were included
- This could be added as supplementary analysis

**Response**:

Thank you for highlighting this important issue. We conducted a sensitivity analysis to assess the impact of these biomarkers, and the results have been added to the revised manuscript:

“Moreover, key prognostic factors, including diabetes history, glycated hemoglobin (HbA1c), and tumor markers such as pre-treatment CEA and CA19-9, have only been recorded in the Taiwan Cancer Registry since 2021 and were unavailable for the main analysis. In a sensitivity analysis of the 2021–2023 cohort (≈90% case coverage; 7,336 patients) using the same cross-validation workflow, adding these biomarkers modestly improved the 1-year AUC from 0.855 to 0.865 and the 3-year AUC from 0.890 to 0.895 (Supplementary Figure S11), suggesting scope for further gains; future studies incorporating these biomarkers alongside genomic features (e.g., KRAS, TP53 mutations) and imaging data may further improve prediction and reveal additional interaction patterns.”

*Minor Issues*

Methods

4. Multiple Imputation Reporting (Lines 138-141)

- Add table showing proportion of missing data for each variable
- Report convergence diagnostics for MICE imputation
- Clarify whether follow-up time and event indicator were used in imputation model (you state they were excluded, which is appropriate—good)

**Response**:

Thank you for your suggestion. We added convergence diagnostics for MICE and the proportion of missing values, as described in the Methods section:

“The MICE algorithm was run for 25 iterations, and convergence was assessed using autocorrelation and the potential scale reduction factor. Autocorrelations approaching zero and scale reduction factors approaching one indicated adequate convergence.^29^ The proportion of missing values for each predictor and the corresponding convergence diagnostics are shown in Supplementary Table S2 and Supplementary Figure S2, respectively”.

Additionally, the follow-up time and event indicator were excluded from the imputation process to avoid risk information leakage from the outcome to the prognostic factors.

5. Calibration Assessment

- The study reports discrimination (AUC, sensitivity, specificity) but not calibration
- Add calibration curves or calibration slope/intercept at key time points (1, 3, 5 years)
- This is important for clinical use where absolute risk estimates matter

**Response**:

Thank you for your suggestion. We added time-dependent calibration curves to assess agreement between predicted risks and observed outcomes, as described in the Results section:

“The calibration curves of all competing models at 1, 3, and 5 years after cancer diagnosis are shown in Supplementary Figure S4, and the corresponding calibration slopes and intercepts indicate that predicted survival closely matches observed outcomes across models”.

6. Hyperparameter Selection (Lines 145-147, Supplementary Table S2)

- Briefly justify the hyperparameter search space ranges
- Report final selected hyperparameters for the best model
- Add computational time information

**Response**:

Thank you for your suggestion. We detailed the rationale for hyperparameter search ranges, and reported the final selected hyperparameters for XGBoost in Supplementary Table S3. The computational time information was mentioned in the Methods section:

“The runtimes for training and hyperparameter tuning of all models in each fold are compared in Supplementary Figure S3”.

Results

7. Figure 1 Performance Plateau (Lines 172-179)

- The plateau after year 3 is mentioned but not explained
- Add brief explanation (e.g., "likely due to most deaths occurring within 3 years and changing censoring patterns thereafter")

**Response**:

Thank you for your suggestion. We added to the Results section:

“Figure 1 shows that performance improves across all models until about 3 years after diagnosis, then plateaus, likely because most deaths occur within the first 3 years (yielding richer signal) while increasing censoring thereafter reduces events for evaluation”.

8. Quantifying Effect Heterogeneity (Lines 221-236, Figure 4)

- While interactions are visualized, provide confidence intervals or bootstrapped uncertainty estimates for key stratified hazard ratios
- This would strengthen claims about differential treatment effectiveness

**Response**:

Thank you for your suggestion. We added to the Methods section:

“We also derived HRs for mortality for each feature from the SHAP values and quantified their uncertainty using 95% confidence intervals (95% CIs) based on 1,000 bootstrap resamples”,

and provided confidence intervals to all hazard ratios estimates in the Results section.

9. Comparison with Prior Models (Lines 238-253)

- Limited quantitative comparison with existing pancreatic cancer prognostic models
- Add table comparing your C-statistic/Brier scores with recently published models (references 7-8, 13-16)
- This contextualizes your model's performance

**Response**:

Thank you for your suggestion. We summarized existing pancreatic cancer prognostic models (references 7-16) in Supplementary Table S3, including their data sources, sample sizes, modeling methods, key variables, and time-dependent AUCs. The comparison was mentioned in the Discussion section:

“In addition, when compared with recent pancreatic cancer prognostic models, our XGBoost model achieved superior AUCs across the 1- to 5-year horizons (Supplementary Table S4)”.

Discussion

10. Mechanistic Context for Non-linear Relationships (Lines 254-260)

- The U-shaped BMI relationship and smoking plateau are interesting but lack biological explanation
- Briefly discuss potential mechanisms:

Low BMI: cancer cachexia, nutritional depletion

High BMI: metabolic dysfunction, inflammation

Smoking plateau: survivor bias, competing risks

**Response**:

Thank you for your suggestion. We added clinical explanations to the revealed nonlinear effects of age, smoking, and BMI on pancreatic cancer survival in the Discussion section:

- “The increase in mortality risk accelerates after age 65, which may relate to declining physiological reserve, accumulating comorbidities, and age-dependent tumor biology.”
- “Mortality risk increases with longer smoking duration but appears to plateau between 10 and 30 years, which may reflect survivor bias and competing risks.”
- “Low BMI may reflect nutrient depletion or cachexia, whereas high BMI is indicative of metabolic dysfunction and chronic inflammation.”

11. External Validation (Lines 276-281)

- Acknowledge that cross-validation provides internal validity but external validation in different populations is needed
- Discuss generalizability to non-Asian populations given potential ethnic/genetic differences

**Response**:

Thank you for your suggestion. We performed temporal validation using recent data, and discussed the need for additional external validation in the Discussion section:

“Finally, external validation in independent cohorts remains essential: temporal validation in 2022–2023 yielded 1-year and 2-year AUCs of 0.858 and 0.878, respectively, but geographic and cross-ethnic validation is still needed to establish generalizability across populations and healthcare settings.”

12. Multiple Testing

- With 12 main features and numerous interactions, multiple testing is a consideration
- Briefly acknowledge this as an exploratory study generating hypotheses for confirmation

**Response**:

Thank you for your suggestion. We addressed this concern as a limitation in the Discussion:

“In addition, because we highlighted top features and interactions from a larger set, the analysis is subject to multiplicity and potential selection effects; thus, these findings should be viewed as exploratory and hypothesis-generating rather than confirmatory.”

Presentation

13. Abstract (Lines 2-29)

- Currently very technical with many specific algorithm names
- Simplify for broader audience: "machine learning and deep learning methods" rather than listing all algorithm names
- Move technical details to methods

**Response**:

Thank you for your suggestion. We simplified the descriptions of models in the Abstract:

“We developed three classes of prognostic models using regression-based, machine learning, and deep learning methods”.

14. Figure 3 Clarity

- Some subplots have overlapping points (especially 3f - Age, 3j - Years of smoking)
- Consider transparency, jittering, or violin plots to show distributions better
- Ensure color schemes are colorblind-friendly

**Response**:

Thank you for this suggestion. To improve the readability of Figures 3 and 4, we increased point transparency, reduced point size, and added slight jittering to better visualize the underlying distributions and overlapping observations. We also updated the color scheme in Figure 1 to be colorblind-friendly. For the SHAP-related figures, we retained the default SHAP palette for consistency with standard SHAP visualizations and ensured sufficient contrast for interpretability.

15. Language/Formatting Issues

- Line 67-68: "7-968" appears to be formatting error
- Line 84, 107, 118: Missing spaces before citations
- Lines 99-100: Missing space "Cancer(AJCC)" should be "Cancer (AJCC)"
- Inconsistent abbreviation formatting: "intelligence(AI)" should have space before parenthesis
- Lines 85-93: Very long sentence; break into 2-3 sentences for readability

**Response**:

Thank you for your suggestion. We have addressed these formatting issues in the revised manuscript.

*Additional Recommendations*

Code and Reproducibility

- Deposit analysis code on public repository (GitHub, Zenodo) with documentation
- Include code for SHAP analysis and visualization
- Provide synthetic/simulated data example for reproducibility
- This addresses the “Partly” rating for data availability while respecting privacy restrictions

**Response**:

Thank you for your suggestion. We released the analysis code and updated the Data Availability Statement:

“The analysis code and a synthetic dataset with similar structure and statistical properties to the real data will be deposited in a public GitHub repository (<https://github.com/darentsai/SurvPancreas>), with permanent identifier upon manuscript acceptance.”

The versions of the software and required packages are documented in the website.

Supplementary Materials

Consider adding:

- Patient flowchart showing exclusion criteria
- Table 1 with baseline characteristics stratified by key variables
- Extended SHAP analysis for additional feature pairs
- Calibration plots
- Sensitivity analyses

**Response**:

Thank you for your suggestion. All of these have been added to the Supplementary Materials and mentioned in the revised manuscript:

- Supplementary Table S2 – Baseline characteristics and outcomes for pancreatic cancer patients
- Supplementary Figure S1 – Study flowchart
- Supplementary Figure S4 – Time-dependent calibration curves
- Supplementary Figure S9 – SHAP dependence plots for additional feature pairs
- Supplementary Figure S11 – Time-dependent AUCs from sensitivity analysis

Future Directions

Briefly discuss:

- Plans for prospective validation
- Potential for incorporating genomic/imaging data
- Development of simplified clinical scoring system
- Implementation strategy for clinical adoption

**Response**:

Thank you for your suggestion. We address these issues in the Discussion section:

- “Our explainable AI model could support clinical decision-making in several ways. First, it may assist risk stratification by identifying patients at elevated risk who could benefit from closer monitoring, earlier integration of palliative care, or consideration of more intensive treatment options. The treatment-related interactions revealed by the model also help characterize how prognosis varies across clinical subgroups. For example, our findings suggest that older patients with advanced-stage adenocarcinoma constitute a particularly high-risk group in whom the mortality reduction associated with chemotherapy appears most pronounced, a pattern that may warrant further investigation in dedicated clinical or observational studies.”
- “Second, patient-level SHAP values enable transparent, individualized risk communication: clinicians can show which factors contribute most to a specific patient’s predicted risk and how changes in key variables (e.g., smoking duration, BMI) might influence prognosis. Supplementary Figure S10 illustrates clinical vignettes for three example patients with distinct characteristics, displaying their input features, corresponding SHAP values, predicted survival curves, and possible clinical interpretation.”
- “Finally, several implementation issues would need to be addressed before clinical deployment, including integration with electronic health record systems, development of user-friendly interfaces, clinician training on model interpretation, and ongoing surveillance for performance drift over time. Together, these elements outline a potential pathway for translating our model into a practical clinical decision-support tool, while recognizing that its role should complement, rather than replace, clinical judgment and guideline-based care.”
- “Moreover, key prognostic factors, including diabetes history, glycated hemoglobin (HbA1c), and tumor markers such as pre-treatment CEA and CA19-9, have only been recorded in the Taiwan Cancer Registry since 2021 and were unavailable for the main analysis. In a sensitivity analysis of the 2021–2023 cohort (≈90% case coverage; 7,336 patients) using the same cross-validation workflow, adding these biomarkers modestly improved the 1-year AUC from 0.855 to 0.865 and the 3-year AUC from 0.890 to 0.895 (Supplementary Figure S11), suggesting scope for further gains; future studies incorporating these biomarkers alongside genomic features (e.g., KRAS, TP53 mutations) and imaging data may further improve prediction and reveal additional interaction patterns.”
- “Finally, external validation in independent cohorts remains essential: temporal validation in 2022–2023 yielded 1-year and 2-year AUCs of 0.858 and 0.878, respectively, but geographic and cross-ethnic validation is still needed to establish generalizability across populations and healthcare settings.”

Minor Editorial Corrections

1. Line 53: "highly developed countries" → "high-income countries" (standard terminology)

2. Line 100: Choose "histological" or "histologic" and use consistently

3. Line 210: Clarify "0.69% per year" vs "0.69 percentage points per year"

4. Ensure consistent citation formatting throughout

5. Abstract line 10-12: Break long sentence listing all algorithms

**Response**:

Thank you for your suggestion. We have addressed all these editorial issues in the revised manuscript. We detailed how mortality risk increases with age, expressed as relative percentage change:

“The slopes of the SHAP values for age are 0.0068 and 0.0238 before and after age 65, respectively, indicating that the hazard of death increases by [exp(0.0068)−1]×100%=0.69% per year before age 65 (95% CI: 0.66%–0.71%), and by [exp(0.0238)−1]×100%=2.41% per year thereafter (95% CI: 2.39%–2.45%).”

The descriptions of models in the Abstract were simplified:

“We developed three classes of prognostic models using regression-based, machine learning, and deep learning methods”.

*Questions for Authors*

1. Have you explored temporal trends by diagnosis year (2013-2021) to assess whether treatment effectiveness has changed?

**Response**:

Thank you for this important suggestion. We agree that treatment–outcome associations may vary over calendar time as staging practices and standards of care evolve. We have acknowledged this in the Discussion:

“Third, we did not include diagnosis year (2013–2021) as a covariate and therefore did not assess whether treatment–outcome associations varied across calendar time; future work could incorporate diagnosis year and evaluate treatment-by-year heterogeneity, including via SHAP interaction patterns, to examine temporal variation in these associations.”

2. How consistent are SHAP values across the 5 imputed datasets?

**Response**:

The consistency of SHAP values has been addressed in the revised manuscript:

“Leveraging XGBoost’s built-in tree-based mechanism for handling missing values, the model was trained and SHAP values were computed directly on the original (non-imputed) data. For comparison, we also trained XGBoost models separately on the five imputed datasets and averaged their SHAP values; the resulting importance rankings were highly consistent with those from the original data, with Kendall’s τ of 0.95 for feature importance and 0.89 for interaction importance. We therefore adopted the non-imputed approach for subsequent analyses, allowing each feature’s effect size to be assessed using SHAP values from patients with complete records for that feature, without reliance on imputed values”.

3. What was the concordance between model predictions and actual treatment decisions?

**Response**:

The time-dependent calibration curves show that the predicted survival from our model closely matches observed outcomes. However, because our model is prognostic rather than prescriptive, it does not generate treatment recommendations, and concordance with clinical treatment decisions was not evaluated. We acknowledged this in the Discussion:

“One key limitation is the observational design, as treatment–outcome associations may be influenced by residual confounding, confounding by indication, and treatment selection bias (e.g., unmeasured differences in performance status, frailty, or disease progression between treated and untreated patients). Accordingly, the observed effects of surgery and chemotherapy represent prognostic associations rather than causal estimates and should be interpreted with caution; these associations should be validated in randomized trials or rigorously controlled observational studies before being used to inform treatment allocation. In addition, because the model is prognostic rather than prescriptive, it does not generate treatment recommendations, and we did not assess concordance between model outputs and clinical treatment decisions.”

4. Did you consider incorporating treatment sequences/timings rather than just binary indicators?

**Response**:

We acknowledged this issue as a limitation in the Discussion section:

“Second, treatments were coded as baseline “ever/never” variables, which may introduce immortal-time bias because patients must survive long enough after diagnosis to receive surgery or chemotherapy; this can overstate apparent associations by attributing pre-treatment survival time to the treated group. Future analyses should model treatments as time-dependent covariates or use landmark analyses to better align exposure definition with clinical timing.”

Conclusion

This is a well-executed study demonstrating how explainable AI can provide clinically interpretable insights for pancreatic cancer prognosis. The methodological rigor is high, and findings are clinically relevant, particularly the treatment-histology interactions. After addressing the issues above—especially clarifying treatment effect interpretation, enhancing clinical utility discussion, and improving reproducibility—this manuscript will make a valuable contribution to PLOS Digital Health. The work successfully moves beyond "black box" AI predictions to provide actionable clinical insights.

Recommendation: Accept with Minor Revisions

**Response**:

Thank you for your positive and constructive comments.

**Reviewer #5**

This is a strong and well-executed study that combines methodological rigor with clinical relevance. The use of a nationwide registry, the comparison between traditional, machine learning, and deep learning survival models, and the integration of SHAP-based explainability make this work both comprehensive and timely. The results are clear, and the interpretability analysis provides meaningful insights into how different clinical and behavioral factors interact to shape patient outcomes in pancreatic cancer.

**Response**:

Thank you for your positive and constructive comments.

That said, a few refinements would strengthen the manuscript before publication. Abstract and methods could be tightened to improve flow and readability. The focus of the abstract could shift slightly toward the explainability outcomes rather than technical descriptions of the models. A bit more detail about the computational environment, software versions, and hyperparameter tuning ranges would enhance reproducibility. Figure readability can also be improved, especially in the SHAP plots, where increasing resolution and defining all abbreviations directly in the legends would make the results more accessible.

The discussion is thoughtful but could more explicitly link the explainable AI framework to clinical practice. A short paragraph on how these insights might translate into clinical decision-support tools, or how future versions could integrate biomarkers like CA19-9, would help emphasize practical utility. Finally, a light editorial review for formatting consistency, reference spacing, and parenthesis placement would bring the paper fully in line with PLOS Digital Health’s stylistic standards.

Overall, this is a valuable and well-written contribution. With these minor revisions for clarity, reproducibility, and presentation, the paper will be in excellent shape for publication.

**Response**:

Thank you for highlighting these important issues.

1. We simplified the descriptions of models in the Abstract for broader readers.
2. We released the analysis code and updated the Data Availability Statement: “The analysis code and a synthetic dataset with similar structure and statistical properties to the real data will be deposited in a public GitHub repository (https://github.com/darentsai/SurvPancreas), with permanent identifier upon manuscript acceptance”. The versions of the software and required packages are documented in the website.
3. We detailed the rationale for hyperparameter search ranges, and reported the final selected hyperparameters for XGBoost in Supplementary Table S3.
4. For SHAP-related plots, we increased transparency, reduced point size, and added slight jittering to continuous features to better recognize the distributions. All abbreviations were listed in the figure legends.
5. We elaborated broadly on clinical applications and implementation considerations in the Discussion section:

- “Our explainable AI model could support clinical decision-making in several ways. First, it may assist risk stratification by identifying patients at elevated risk who could benefit from closer monitoring, earlier integration of palliative care, or consideration of more intensive treatment options ……”
- “Second, patient-level SHAP values enable transparent, individualized risk communication: clinicians can show which factors contribute most to a specific patient’s predicted risk and how changes in key variables (e.g., smoking duration, BMI) might influence prognosis ……”
- “Finally, several implementation issues would need to be addressed before clinical deployment, including integration with electronic health record systems, development of user-friendly interfaces, clinician training on model interpretation, and ongoing surveillance for performance drift over time ……”

6. We conducted the sensitivity analysis to evaluate the impact of missing biomarkers (CA19-9 etc.). The results suggested scope for further performance gains.
7. We have addressed these formatting issues in the revised manuscript.
